# Challenges and Recent Advances in NK Cell-Targeted Immunotherapies in Solid Tumors

**DOI:** 10.3390/ijms23010164

**Published:** 2021-12-23

**Authors:** Guangyu Lian, Thomas Shiu-Kwong Mak, Xueqing Yu, Hui-Yao Lan

**Affiliations:** 1 Guangdong-Hong Kong Joint Research Laboratory on Immunological and Genetic Kidney Diseases, Department of Pathology, Guangdong Provincial People’s Hospital, Guangdong Academy of Medical Sciences, Guangzhou 510080, China; gylian@cuhk.edu.hk; 2 Guangdong Cardiovascular Institute, Guangdong Provincial People’s Hospital, Guangdong Academy of Medical Sciences, Guangzhou 510080, China; 3 Department of Medicine & Therapeutics, Li Ka Shing Institute of Health Sciences, The Chinese University of Hong Kong, Hong Kong SAR, China; thomak@hotmail.com.hk

**Keywords:** NK cell, immunotherapy, solid tumor, immune checkpoint inhibitors, BiKE, TriKE, CAR-NK, NK cell therapy

## Abstract

Natural killer (NK) cell is a powerful malignant cells killer, providing rapid immune responses via direct cytotoxicity without the need of antigen processing and presentation. It plays an essential role in preventing early tumor, metastasis and minimal residual disease. Although adoptive NK therapies achieved great success in clinical trials against hematologic malignancies, their accumulation, activation, cytotoxic and immunoregulatory functions are severely impaired in the immunosuppressive microenvironment of solid tumors. Now with better understandings of the tumor evasive mechanisms from NK-mediated immunosurveillance, immunotherapies targeting the key molecules for NK cell dysfunction and exhaustion have been developed and tested in both preclinical and clinical studies. In this review, we introduce the challenges that NK cells encountered in solid tumor microenvironment (TME) and the therapeutic approaches to overcome these limitations, followed by an outline of the recent preclinical advances and the latest clinical outcomes of NK-based immunotherapies, as well as promising strategies to optimize current NK-targeted immunotherapies for solid tumors.

## 1. Introduction

As a central part of the innate lymphoid cells (ILCs), NK cells are cytotoxic large granular lymphocytes capable of killing tumor cells and viral-infected cells without the prerequisite of priming [1]. Being part of the innate immune system, NK cells serve as the first line of defense against hematologic and solid malignancies via prompt recognition of malignant cells during carcinogenesis, preventing metastasis and clearing minimal residual disease [2,3,4,5].

Remarkable progress in immunotherapeutic research, including bispecific antibodies, immune checkpoint inhibitors (ICIs) and chimeric antigen receptor (CAR) T cells, has achieved tremendous success from bench to bedside in recent decades [6,7]. Compared with T cells, NK cells have advantages of superior feasibility, lower risk of side effects, faster response, and stronger cytokine release capacity to communicate with other immune cells [8,9,10]. Therefore, the emerging NK-targeting immunotherapies provide alternative approaches for cancer patients who are not suitable for T cell-based therapies.

However, despite the great success of NK adoptive immunotherapy achieved in hematologic cancers, the microenvironment of solid cancers severely blunts NK-mediated cytotoxicity by reducing infiltration, impairing target cells recognition, suppressing activation, as well as weakening immunoregulatory and cytotoxic functions of NK cells [11,12]. Therefore, restoring NK-mediated immunosurveillance would provide a promising therapeutic target for patients suffering from solid tumors.

Currently, NK cells-targeting immunotherapies, including the traditional cytokine administration, ICIs, bi-specific or tri-specific killer cell engagers (BiKEs or TriKEs), and the more recently developed genetically modified NK cells such as CAR-NKs, have been exploited and optimized to restore NK immunity in the immunosuppressive microenvironment [13,14]. Here, we specifically analyze the challenges and therapeutic strategies for NK recruitment, recognition, activation and function in solid tumors, while evaluating the advantages and disadvantages of different categories of immunotherapies.

## 2. NK Cell Biology

### 2.1. Subtypes of NK Cells

Human NK cells are categorized according to the level of CD56. The CD56^bright^ population is precursor NK with lower cytotoxicity and higher capacity of cytokine secretion, whereas CD56^dim^ is terminally matured NK with higher cytotoxicity predominating in peripheral blood [15,16,17]. In mice, the development of NK cells is divided into four stages, in sequence of CD11b^low^CD27^low^, CD11b^low^CD27^high^, CD11b^high^CD27^high^, and finally CD11b^high^CD27^low^, among which CD11b^high^CD27^high^ NK cells possess the most effective killing capacity [18]. Importantly, the expression level of CD27 is also correlated with the cytotoxicity of human NK cells, indicating CD27^high^ human NK cells are more potent than CD27^low^ NK cells in cytotoxicity [19,20].

Apart from conventional NK cells (cNKs) circulating in peripheral blood, tissue-resident NK cells (trNKs) are also identified in liver, lung, uterus, lymph node, thymus, and tumor tissue [21]. These two subsets of NK cells were defined with the levels of CD49b and DX5, with CD49a^−^DX5^+^ NK as circulating cNK and CD49a^+^DX5^−^ NK as trNK [22]. The majority of cNK cells in peripheral blood are CD56^dim^, while resident NK cells in lymphoid tissues, uterus, and liver are mostly CD56^bright^ [21,23].

### 2.2. Chemotaxis of NK Cells

**Figure 1:**
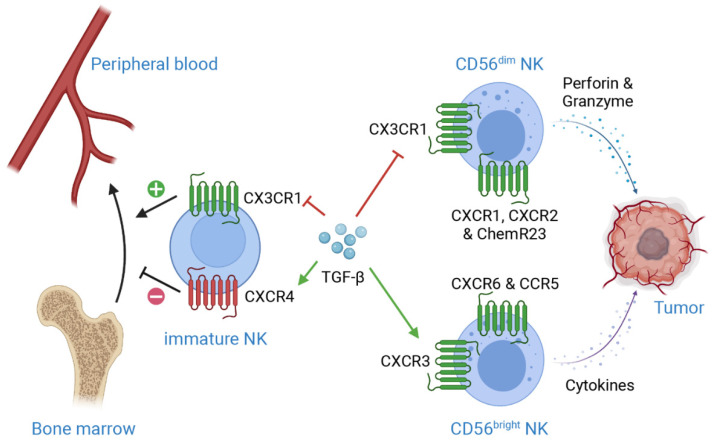
**Mechanisms of TGF-β-induced suppression on NK cell infiltration in TME.** CXCR4 is essential for NK development and retention in bone marrow, while CX3CR1 plays important roles in NK egression from bone marrow. TGF-β enhances CXCR4 level but downregulates CX3CR1 level on immature NK cells, which results in retention of immature NK cells in bone marrow, subsequently preventing NK maturation. Moreover, TGF-β induces the expression of CXCR3 on CD56^bright^ NK cells, while suppresses the expression of CX3CR1 on CD56^dim^ NK cells. These regulations lead to the accumulation of CD56^bright^ NK, a less cytotoxic phenotype as compared to CD56^dim^ NK, in TME. Additionally, CXCR1, CXCR2 and ChemR23 are crucial for CD56^dim^ NK recruitment, while CXCR6 and CCR5 are important for CD56^bright^ NK recruitment. This figure is created with biorender.com.

Peripheral blood NK cells migrate into organs and tumors in response to diverse chemokines. As mentioned previously, the phenotype and functions of CD56^dim^ cNKs are very different from CD56^bright^ trNKs, as are their expressions of chemokine receptors. CD56^dim^ NKs uniquely express CX3CR1, CXCR1, CXCR2 and ChemR23, while CD69, CXCR3, CXCR6 and CCR5 are commonly found in CD56^bright^ NK cells [24,25] (Figure 1). The expression of CCR7 is responsible for the recruitment of circulating NK cells to secondary lymphoid tissue, while high level of CXCR3 is detected on tumor-infiltrating NK cells [26,27,28].

### 2.3. Activation of NK Cells

The activation of NK cells is under strict and complex regulations by germline-encoded receptors that can be divided into two subsets according to their functions: the activating and inhibitory receptors. The equilibrium of activating and inhibitory signals determines whether NK cell will kill its target or not.

Type I transmembrane receptors natural cytotoxicity receptors (NCRs) are one of the first identified activating receptors for NK cells, members including constitutively expressed NKp46 and NKp30, as well as NKp44, which is upregulated upon stimulations of IL-2, IL-15 or IL-1β. NCRs can directly induce NK cell-mediated cytotoxicity and cytokine secretion when they bind to ligands expressed on tumor cells or viral infected cells [29].

The lectin-like type 2 transmembrane receptor NKG2D is another crucial activating receptor. It functions through interacting with adaptor protein DAP10, which subsequently initiates cytotoxic functions and cytokine release via PI3K signaling [30]. NKG2D is activated by a wide range of ligands, including MHC class I chain-related protein A and B (MICA and MICB), as well as ULBP families in humans, and H60, RAE and MULT1 families in mice [31].

The Ig-superfamily receptor DNAM-1 (CD226) also plays an important role in NK cell activation as methylcholanthrene-induced fibrosarcoma shows largely increased progression in DNAM-1-deficient mice [32]. DNAM-1 recognizes two ligands belonging to the Nectin family, CD155 (PVR) and CD112 (Nectin-2), which are widely expressed on various tissues and cancers. The activation of DNAM-1 stabilizes the adhesion of NK cells to target cells via interacting with LFA-1. More importantly, it promotes NK-mediated cytotoxicity and cytokine release via interacting with Grb2 in the presence of an immunoreceptor tyrosine tail (ITT)-like motif [33,34].

Another mechanism that can directly initiate NK cell-mediated cytotoxicity is antibody-dependent cell cytotoxicity (ADCC). Human NK cells recognize the Fc portion of human immunoglobulins that are expressed on target cells via FcγRIIC (CD32c) or FcγRIIIA (CD16a), which subsequently trigger cytotoxic activity of NK cells against these target cells [10].

NK cells recognize aberrant cells that are absent of major histocompatibility complex (MHC) class I molecules via its inhibitory receptors, providing the “license” for natural killing. This mechanism is called “missing self”, which secures healthy “self” cells expressing MHC I molecules from the attack of NK cells [35,36]. The inhibitory receptors include killer cell immunoglobulin-like receptors (KIRs) (only in humans), LY49 family (only in mice), and CD94-NKG2A (in both humans and mice) [37,38]. CD94/NKG2 family receptors are heterodimer receptors consisting of CD94 and one of the NKG2 family receptors, namely NKG2A, NKG2B, NKG2C, NKG2E or NKG2H. The CD94/NKG2 receptors recognize HLA-E in humans, and MHC class Ib molecule Qa1 in mice [39,40]. In fact, CD94/NKG2A is the first receptor expressed on immature NK cells. During maturation, NK cells will gradually lose NKG2A, but acquire KIRs on the contrary. KIRs are immunoglobulin superfamily receptors including both activating and inhibitory receptors, and each member recognizes a subgroup of HLA class I allotype [41,42]. The expression of KIRs among different NK cell clones seems to be random, except for KIR2DL4 which expresses on all NK cells [43]. All mature human NK cells express either KIRs or NKG2A to avoid the presence of autoreactive NK cells via a SHP-1-mediated mechanism [41,44]. In correspondence, Ly49 plays a similar role in mice as KIRs in humans [45].

### 2.4. The Killing Effect of NK Cells

When an NK cell is activated, it will form an immunological lytic synapsis with the target cell via firm cell-cell adhesion. Then, perforin is secreted from cytoplasmic granules to permeabilize membrane of the target cell and induce osmotic lysis, accompanied by the exocytosis of granzymes to induce apoptosis of the target cell [46]. NK cells also kill target cells via expressing death-inducing ligands, such as FAS ligand (FasL) and TNF-related apoptosis-inducing ligand (TRAIL). The binding of these ligand with corresponding receptors, FAS and DR4/DR5, will initiate death receptor-mediated apoptosis in target cells [47].

As one of the essential parts of cancer immunosurveillance in the early stage, NK cells recruit other hematopoietic cells via releasing chemokines such as CCL3, CCL4, CCL5, CXCL2, CXCL3 and CXCL7. Meanwhile, it exerts immunoregulatory functions by releasing cytokines such as IFN-γ, IL-10, TNF and GM-CSF [48,49,50]. NK cell is one of the major sources of IFN-γ. As a potent immunoregulatory cytokine, IFN-γ enhances the level of MHC class I on tumor cells as well as the expression of MHC class II on antigen presenting cells, thus facilitating antigen presentation and T cell activation [50,51,52].

## 3. NK Cells Immune Response in Solid Tumor Microenvironment

### 3.1. Importance of NK-Mediated Cancer Surveillance

Numerous studies have evidenced the importance of NK cells in the defense against various types of cancer in the last 40 years [53]. Depletion of NK cells with antibodies or impairing their cytotoxic functions leads to tumor growth and metastasis [54,55,56]. O’Sullivan et al. [57] identified in the absence of adaptive immunity in the Rag^-/-^ mice, immunosurveillance mediated by NK cells and macrophages can prevent methylcholanthrene-induced sarcomas. Consistently, clinical studies also proved the correlation of higher number of tumor-infiltrated NK cells with better prognosis in solid cancers, including lung cancer, liver cancer, head and neck cancer, breast cancer and colorectal cancer [58,59]. An 11-year follow-up study reported that low cytotoxic activity of NK cells is associated with higher risk of cancer incidence, illustrating the pivotal significance of NK-mediated cytotoxicity in cancer immunosurveillance [60]. Recent studies also showed that higher expressions of activating receptors on NK cells are associated with improved outcomes in patients with breast cancer and prostate cancer [61,62].

Functionally, NK-derived IFN-γ also induces the polarization and activation of M1 macrophages, which kill tumor cells together with the activated NK cells [57]. IFN-γ also promotes the recruitment of cytotoxic CD27^high^ NK cells in a CXCR3-dependent manner through upregulating the production of CXCL9 and CXCL10 by tumor cells [28]. More importantly, though previously recognized as short-lived cytotoxic killer cells, NK cells are recently identified with antigen-specific memory characteristics like T cells and B cells, which provides an NK-mediated immunosurveillance for up to 3 months [63,64,65].

### 3.2. Challenges for NK-Mediated Immunosurveillance in Solid Tumors

Despite the success of NK cell-based therapies in hematologic malignancies, their efficacy in solid tumor is largely limited. Mechanistically, these suboptimal results may be attributed to the immunosuppressive solid tumor microenvironment, which largely debilitates the infiltration, recognition, activation and effector functions of NK cells [66].

#### 3.2.1. Limiting NK Cell Infiltration

In spite of the strong cancer killing capacity, NK cells are scarcely distributed in solid malignancies such as non-small cell lung carcinoma (NSCLC) [67], hepatocellular carcinoma [68], gastric cancer [69] and colorectal cancer [70,71]. This scarcity of infiltration may be attributed to the decreased expressions of CCL27, CXCL12 and CCL21, which are crucial for NK cell recruitment [72]. Notably, the expressions of CXCR5 and CXCR6 are elevated, while CX3CR1 and S1PR1 are decreased in tumor-infiltrated NK cells in lung carcinoma and neuroblastoma. Hence, the less cytolytic CD56^bright^ NK cells are preferentially recruited in these TMEs [73,74] (Figure 1). Additionally, tumor resident CD103^+^ NK cells express more inhibitory receptors, such as TIGIT and TIM-3, as compared with recruited NK cells, indicating the undermined NK-mediated immunosurveillance in TME [72].

TGF-β, a prominent immunosuppressive cytokine highly enriched in solid tumor microenvironment, significantly enhances the expression of CXCR3 and CXCR4, while inhibits CX3CR1 in human NK cells. These, in turn, impede the egress of NK cells from bone marrow, suppress their maturation consequently, and hence impair the migration of highly cytotoxic CD56^dim^ NKs to TME [74,75] (Figure 1). The deficiency of NK cells in solid tumors is also associated with the accumulation of hyaluronan, which induces a high-pressure environment and leads to vascular collapse, therefore limiting immune cell infiltration into solid tumors [76].

#### 3.2.2. Disrupting NK Recognition and Activation

Cancer cells also escape from NK cell-mediated immunosurveillance via hampering NK recognition of target cells and dampening their activation. TGF-β is largely produced by tumor cells, regulatory T cells (Tregs) and myeloid derived suppressor cells (MDSCs) in the TME, and it has a strong association with poor prognosis in lung carcinoma [77], pancreatic cancer [78], gastric cancer [79], colorectal cancer [80], and hepatocellular carcinoma [81]. It largely impairs the recognition and activation of NK cells via downregulating the expression of NKp30 and NKG2D [82,83]. In contrast, neutralizing TGF-β1 with monoclonal antibody (mAb) can completely restore the expression of NKG2D, as well as the cytotoxic functions of NK cells [84].

Besides, tumor cells can shed their own NKG2DLs, either with metalloproteinases or via exosome secretion, to evade NK recognition via NKG2D [85,86]. This results in high levels of soluble NKG2DLs, such as MICA, MICB and ULBP, which are associated with weakened T and NK functions, and poor prognosis in melanoma, ovarian cancer and prostate cancer [87,88,89]. Meanwhile, tumor cells also induce NKG2DLs expression on healthy myeloid cells through secreting lactate dehydrogenase 5 (LDH5) [90]. The long-term chronic stimulation of NKG2DLs will eventually downregulate the expression of NKG2D on effector NK cells, thereby impeding NK cell activation and diminishing its effector functions [91,92].

**Figure 2:**
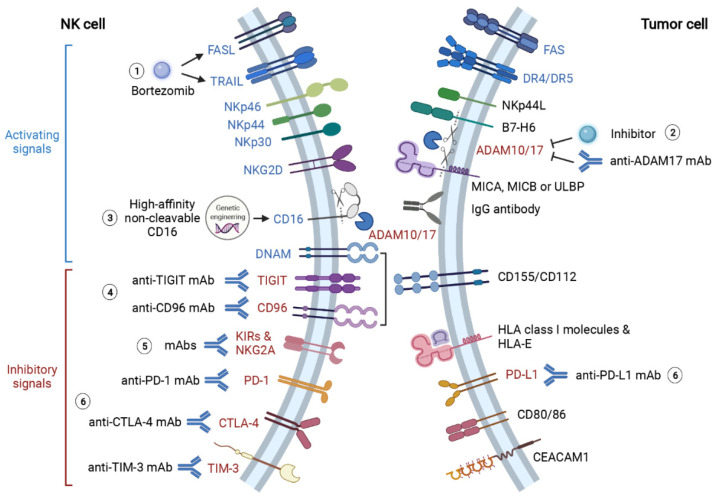
**Summary of therapeutic approaches to reinforce NK recognition and activation in solid tumor microenvironment.** TME blunts NK recognition of tumor cells via diverse mechanisms. The key mediators of these mechanisms represent promising therapeutic targets to restore NK recognition and activation. (**1**) Proteasome inhibitor bortezomib augments NK anti-cancer effects by enhancing TRAIL- and FasL-mediated cytotoxicity. (**2**) NK recognition of tumor antigens is significantly hampered by protease ADAM10- and ADAM17-mediated shedding of CD16, B7-H6 and NKG2DL (MICA, MICB and ULBP). Thereby, treatments of anti-ADAM17 mAb and ADAM inhibitors can promote NK recognition and activation via CD16, NKp30 and NKG2D. (**3**) Additionally, the genetic-engineered “off-the-shelf” NK with high-affinity non-cleavable CD16 (FT516) is also resistant to protease-mediated cleavage, and its therapeutic effect is now being tested in clinical trials. (**4**) Activating receptor DNAM competes for ligands CD155 and CD112 with inhibitory receptors TIGIT and CD96. Monoclonal antibody therapies targeting TIGIT and CD96 effectively inhibit cancer progression and metastasis by preventing TIGIT- and CD96-induced NK exhaustion. (**5**) KIRs and NKG2A play crucial roles in the “missing self” mechanism of NK cells. However, tumor cells take advantage of this mechanism to evade NK-mediated immunosurveillance. Blocking these inhibitory signals with monoclonal antibodies also restores NK cytotoxicity against tumor cells. (**6**) Immune checkpoint molecules PD-1, PD-L1, CTLA-4 and TIM-3 induce NK cell exhaustion when they bind to corresponding ligands on tumor cells. ICIs targeting these molecules largely enhance NK recognition and cytotoxicity against tumor cells. This figure is created with biorender.com.

Apart from modulating the expression of activating receptors on NK cells, tumor cells also evade NK-mediated immunosurveillance by enhancing inhibitory signalings, including inhibitory receptors KIRs and NKG2A, as well as immune checkpoints programmed cell death 1 (PD-1), lymphocyte-activation gene 3 (LAG-3), CD96 (also known as TACTILE), T cell immunoglobulin and ITIM domain (TIGIT), T cell immunoglobulin and mucin-domain containing-3 (TIM3), cytotoxic T-lymphocyte-associated protein 4 (CTLA-4) and Interleukin-1 receptor 8 (IL1R8) [66,93] (Figure 2). As NKG2A is expressed on most tumor-infiltrated NK cells, the level of HLA-E, the ligand for NKG2A, is usually overexpressed in several tumors to induce inhibitory signals to NK cells [94,95,96]. Similar to cytotoxic T cells, the expression of both PD-1 and LAG-3 induces NK cells exhaustion, with LAG-3 upregulated in the early stage and PD-1 increased in terminal stage of exhaustion respectively [97,98,99]. CD96 and TIGIT share the same ligand CD155 and compete for it with activating receptor DNAM-1 [100]. Meanwhile, CD96 and TIGIT are associated with NK cell exhaustion by blunting its cytotoxicity [101,102]. Compared with circulating cNKs, CD49a^+^ trNKs express more inhibitory receptors including PD-1, CD96 and TIGIT. The higher proportion of CD49a^+^ NK in tumor-infiltrated NKs is correlated with lower survival rate in patients with hepatocellular or bladder urothelial carcinoma [103]. Similarly, the presence of a recently identified CD73^+^ NK cells, characterized by high levels of LAG-3, PD-1 and PD-L1, is also associated with poor prognosis in breast cancer patients, evidencing the immunosuppressive role of these immune checkpoint molecules [104]. Another two checkpoints shared by T cell and NK are TIM-3 and CTLA-4. Albeit highly expressed on all matured NK cells, TIM-3 induces NK cell exhaustion when cross-linked with cognate ligands expressed on tumor cells [105]. And high-level TIM-3 expression is correlated with poor prognosis in melanoma patients [106]. As an essential checkpoint for cytotoxic T cell activation, CTLA-4 also represses NK cell proliferation and IFN-γ secretion upon binding to CD80 and CD86 [107,108]. IL1R8 (also known as SIGIRR), the co-receptor for IL-37, is a recently identified checkpoint molecule on NK cells. Deficiency of IL1R8 is correlated with enhanced expression of NKG2D, higher cytotoxicity and improved productions of IFN-γ and granzyme B [109].

As one of the major source of TGF-β in TME, platelets are found to facilitate the evasion of tumor cells from NK immunosurveillance by impairing NK recognition of tumor cells [83,110]. Placke et al. [111] discovered platelets can transfer their normal MHC-I molecules to tumor cells, converting tumor cells into a “pseudonormal” phenotype, which assists tumor cells to escape from both T cell- and NK cell-mediated immunosurveillance.

#### 3.2.3. Impairing NK Effector Function

The TME contains a large number of immunosuppressive molecules, including TGF-β, IL-10, prostaglandin E2, indoleamine 2,3-dioxygenase, adenosine and lactic acid. The majority of them are produced by tumor cells, stroma cell and regulatory immune cells. Specifically, the activation of TGF-β signaling induces the conversion of effector NK cells to more dormant ILC1s phenotype, thus facilitating the growth and metastasis of tumor [112]. TGF-β is also found to suppress the metabolic activity, thus hampering the development and effectiveness of NK cells in response to IL-2 and IL-15 [113,114]. Correspondingly, tumor-induced fructose-1,6-bisphosphatase (FBP1) expression also results in NK cell dysfunction via metabolic suppression [115]. In addition, high level of IL-6 in the endometrial TME also contributes to the malignancy of tumor by suppressing NK cytotoxicity through JAK/STAT pathways [72].

Apart from cell components, cytokines and chemokines, the metabolic environment also expedites tumor growth and metastasis. For instance, the aberrant microvasculature in solid tumor results in a hypoxic TME, which limits NK cytotoxicity via inhibiting the expressions of NKp46, NKp30, NKp44 and NKG2D, as well as the productions of granzyme B and perforin [116,117]. Even though intratumoral NK cells can adapt to hypoxic TME by upregulating HIF-1α, its expression is associated with suppressed activation and decreased IFN-γ production [118]. Meanwhile, the nutrient-deficient environment has metabolically restrictive influences on NK cells, thus significantly impairing their anti-cancer effects. Interestingly, CD56^bright^ NK cells are more sensitive to metabolic restriction compared with CD56^dim^ NK cells, since CD56^bright^ NKs require more energy to support IFN-γ production [119]. While CD56^dim^ NK cells are more susceptible to hydrogen peroxide-induced apoptosis and impairment of ADCC in gastric and esophageal cancer [120]. Low pH environment also induces mitochondrial dysfunction and apoptosis in intratumoral NK cells [121]. Moreover, indoleamine 2,3-dioxygenase (IDO) also significantly reduces NK responsiveness. IDO is a tryptophan-catabolizing enzyme converting tryptophan into immunosuppressive catabolites _L_-kynurenine, picolinic acid and quinolinic acid, all of which inhibit T cell and NK cell proliferation in the absence of tryptophan [122]. _L_-kynurenine can further impair NK recognition and activation through NKp46 and NKG2D [123]. Consistent with these findings, high IDO expression is inversely correlated with progression-free survival, as well as T cell and NK cell recruitment, in patients with endometrial cancer [124]. Finally, high concentration of lactate reduces NK effector functions by downregulating the expression of NKp46 and suppressing IFN-γ production [125,126]. Other immunosuppressants that severely blunt NK functions in the TME include prostaglandin E2 [127] and nitric oxide [128].

## 4. Immunotherapies for Restoring NK-Mediated Immunosurveillance in Solid Tumors

Based on the current knowledge of how tumor cells evade NK-mediated immunosurveillance, researchers have developed several types of therapies, including cytokines, immune checkpoint inhibitors (ICIs), NK cell engagers and NK cell adoptive transfer therapies, to reinforce NK infiltration in tumor tissue, improve NK recognition and activation, and also potentiate its cytotoxic effects [14].

### 4.1. Enhancing NK Infiltration

The most effective strategy to promote NK accumulation in solid tumors is to manipulate the expression of chemokine receptors on NK cells. Enhancing CXCR1 through genetic engineering increases NK accumulation in xenograft ovarian cancer without influencing its cytotoxicity [129]. Overexpression of CXCR4 not only improves NK accumulation in glioma, but also further strengthens NK activation and degranulation through Akt and ERK1/2 pathways [130]. In a similar fashion, overexpressing CXCR2 in NK cells ex vivo largely promotes NK homing and adhesion to tumor cells, as evidenced in renal cell carcinoma patients receiving adoptive NK transfer [131]. As the expressions of CCR2 and CCR7 are crucial for NK cell homing and recruitment, these chemokine receptors also serve as potential therapeutic targets to enhance NK cell infiltration in TME [132] (Figure 1).

Meanwhile, increasing chemokines secretion, including CCL3, CCL5, CCL19 and CCL20, from tumor cells also considerably contributes to the recruitment of CD4^+^, CD8^+^ T cells and NK cells in solid tumors [133,134,135,136]. Notably, higher expression of CCL5 is correlated with increased level of NKp46 and longer survival in melanoma patients [134]. Furthermore, overexpression of CXCL10 by local injection of IFN-γ can also increase the accumulation of CXCR3-positive NK cells in melanoma [137]. However, apart from cytotoxic T cells and NK cells, CCL2 can also recruit tumor-associated macrophages, which may contribute to cancer immunoevasion and metastasis [138]. Similarly, CCL5 is capable of inducing Treg cell infiltration in the TME, as well as stimulating melanoma and prostate cancer cell proliferation [138,139,140]. Therefore, further studies are needed to evaluate the potential benefits and risks of chemokine therapies in different types of cancer.

### 4.2. Boosting NK Cell Recognition and Activation

#### 4.2.1. Therapies Targeting Activating Signalings

As an essential component for NK-mediated ADCC, CD16 shedding induced by ADAM17 significantly impedes the effector functions of NK cells [141]. ADAM10 and ADAM17 additionally dampen NK recognition and activation via shedding B7-H6 (a ligand of NKp30) from NK cells, as well as MICs (ligands of NKG2D) from tumor cells [142,143,144]. Blocking ADAM17 with either human mAb MEDI3622 or inhibitor INCB7839 significantly attenuates CD16 shedding and promotes IFN-γ and TNF-α productions by NK cells [141,145] (Figure 2). de Andrade et al. [146] successfully prevented the loss of MICA and MICB on human cancer cells with antibodies targeting MICA α3 domain, the site where MICs are cleaved by proteases. This antibody therapy significantly promotes NK-mediated immunity and suppresses melanoma lung metastasis in humanized mouse models [146]. Furthermore, an iPSC-derived “off-the-shelf” NK therapy (FT516) engineered with high-affinity non-cleavable CD16, which is resistant to ADAM10 and ADAM17 cleavage, is now entering clinical trials in ovarian cancer and other advanced solid tumors (Table 2).

#### 4.2.2. Therapies Targeting Inhibitory Signalings

The discovery of immune checkpoint and the subsequent development of ICIs are revolutionary milestone in solid tumors treatment in the recent decade. The celebrated success is also endorsed with the 2018 Nobel Prize in Medicine, which is jointly awarded to James Allison and Tasuku Honjo, who identified CTLA-4 and PD-1 respectively [147]. Originally discovered in cytotoxic T cells, immune checkpoints molecules are important inhibitory regulators to provide counterbalance to activation signals and to maintain self-tolerance. However, tumor cells take advantage of this inhibitory mechanism to evade immunosurveillance via inducing CD8^+^ T cell exhaustion. As mentioned earlier, recent research also discovered immune checkpoints-mediated inhibitory regulations in NK cells [148]. Thereby, immune checkpoint inhibitors may serve as potential therapies for enhancing NK cell immunity (Figure 2).

##### KIRs and NKG2A Inhibitors

As previously discussed, KIRs and NKG2A limit the effector functions of alloreactive NK cells against cancer. Phase 1 studies of IPH2101, a human IgG4 mAb against KIR2DL1, KIR2DL-2 and KIR2DL-3, illustrated that blocking KIRs with IPH2101 enhances NK cell cytotoxicity against acute myeloid leukemia (AML) and multiple myeloma (MM) without eliciting autoimmune response. Although initially there is safety concern that the use of KIR inhibitors may enable NK cells to attack autologous normal cells, clinical results showed that the therapy is well-tolerated even at the highest dose of 3 mg/kg [149,150]. However, Carlsten et al. [151] pointed out that IPH2101 treatment reduces NK-mediated immune response due to the loss of KIR2D via trogocytosis, and thus failed to generate clinical benefits in MM patients accordingly.

Given the failure of immunotherapies targeting KIRs, the other inhibitory receptor NKG2A may represent as an alternative therapeutic target. The use of monalizumab, a humanized IgG4 anti-NKG2A antibody, can promote NK cell cytotoxicity against HLA-E^+^ target cells with enhanced IFN-γ production. More importantly, monalizumab may function in a synergistic way with durvalumab, an anti-PD1 mAb, and cetuximab, an anti-EGFR mAb, to further improve NK cell- and T cell-mediated immune responses [96]. Notably, blocking inhibitory receptors with antibodies further activates NK-mediated cytotoxicity via ADCC [148]. The efficacy and safety of NKG2A blockade either in monotherapy or in combination with other ICIs in different types of cancer are being tested in registered clinical trials (Table 1) [152].

##### Immune Checkpoint Inhibitors

Due to the revolutionary success of anti-PD-1 and anti-PD-L1 mAb achieved in various solid tumors, the influence of PD-1 and PD-L1 blockade on NK cells is better studied than other inhibitory receptors. The expression of PD-1 is markedly enhanced in tumor-infiltrated NK cells, which severely impairs their cytotoxicity. Meanwhile, NK cell-mediated immunosurveillance is essential for the success of PD-1 and PD-L1 blockade. This is evidenced by the diminished anti-cancer effects of anti-PD-1 or anti-PD-L1 mAb therapies after NK cells depletion [153]. The great importance of NK cell in anti-PD-1 or anti-PD-L1 immunotherapies is also partially attributed to NK-mediated ADCC [154].

As a relatively recent identified immune checkpoint, CD96-targeted therapy for cancer is only evaluated in preclinical studies for the time being. Treatment with anti-CD96 mAb can inhibit the progression and metastasis of melanoma, lung carcinoma and prostate carcinoma through promoting NK cell anti-cancer activity in an IFN-γ-dependent manner. When it is used in combination with anti-PD-1 and anti-CTLA-4 mAb, there is a further increase in NK cell infiltration and IFN-γ production in metastatic melanoma model [155].

Blockade of immune checkpoint molecule TIGIT unleashes NK cells from functional exhaustion and effectively inhibits cancer progression and metastasis in several animal models, including colon cancer, breast cancer and melanoma. More importantly, TIGIT deficiency in NK cells also correlates with enhanced CD226 level and reduced CD96 level, indicating TIGIT is crucial for NK exhaustion via regulating CD226 and CD96 expressions [102]. The clinical benefits of blocking TIGIT is currently under investigation in several clinical trials (Table 1).

Likewise, preclinical study also shows that blocking TIM-3 largely enhances NK proliferation and cytotoxicity against melanoma cells, as well as its IFN-γ-production [106]. There are multiple ongoing clinical trials under active investigation for the therapeutic effects of anti-TIM-3 mAb, whether as monotherapy or as combination therapy with other ICIs in different advanced solid tumors, including lymphoma, liver cancer and melanoma (NCT03489343, NCT03708328, NCT03680508, NCT04139902 and NCT04931654).

LAG-3 is another important checkpoint molecule for the regulations of T and NK cell activation and function via binding to MHC class II molecules. Recent studies illustrated that LAG-3 can physically interact with PD-1 to synergistically exert a potent inhibitory effect on immune responses [156,157]. Preclinical results showed blocking LAG-3 promotes the infiltration and activation of CD8^+^ T cells, but the effects on NK cells remain unclear [158]. Clinical trials targeting LAG-3, as well as in combination with PD-1, are being tested in various solid tumors in clinical studies (Table 1).

#### 4.2.3. Promoting NK Engagement with Tumor Cells

**Figure 3:**
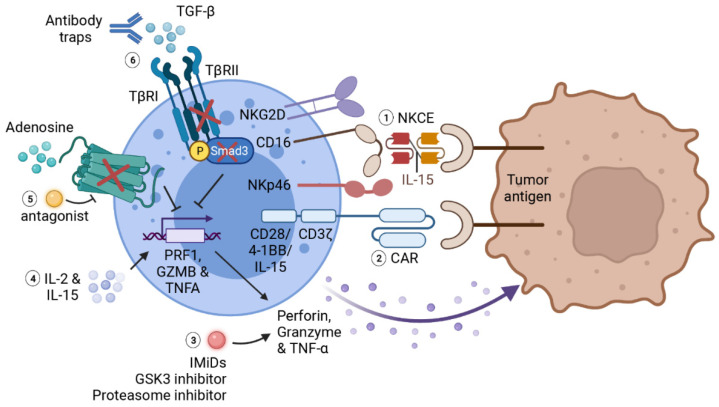
**Summary of immunotherapies for potentiating NK effector functions.** Various therapies have been developed to promote NK effector functions against cancer. (**1**) NKCE facilitates NK recognition of tumor antigens and stabilizes NK adhesion to tumor cells. The addition of IL-15 as a linker further promotes the activation and cytotoxic functions of NK cells. (**2**) Chimeric antigen receptor consists of a scFV of tumor antigen as extracellular antigen-binding domain, a transmembrane domain, and a signaling co-stimulatory domain including CD3ζ, CD28 or 4-1BB. IL-15 gene can also be incorporated into a CAR gene to enhance the survival as well as effector functions of CAR-NKs. The expression of CAR effectively strengthens NK recognition, activation and cytotoxicity in the solid tumor microenvironment. (**3**) Immunomodulatory therapies including IMiDs, GSK-3 inhibitors and proteasome inhibitors can also boost NK-mediated cytotoxicity by increasing cytokine productions by NK cells. (**4**) Cytokine therapies such as IL-2 and IL-15 can largely promote NK activation and cytotoxic functions. However, the severe effects caused by systemic administration of these cytokines limit their application as monotherapies. (**5**) Adenosine is notorious for facilitating tumor evasion from NK-mediated immunosurveillance. Blocking adenosine signaling by genetic depletion of A2A receptor or with adenosine antagonist also augments NK cytotoxic function and cytokine production. (**6**) Impairing TGF-β signaling in NK cell, either with antibody traps, genetical depletion of TGF-β receptor 2 (TβRII) or downstream transcription factor Smad3, can prevent the inhibitory effects of TGF-β on NK activation and function. This figure is created with biorender.com.

Tumor cells use multiple strategies to evade NK-mediated immunosurveillance, including shedding ligands for activation receptors and increasing ligands for inhibitory receptors of NK cells. To overcome these barriers, researchers developed NK cell engager (NKCE) to facilitate the recognition of target cells and to increase adhesion of tumor-infiltrated NK cells onto targeted tumor cells. NKCEs are usually bi-specific killer engagers (BiKEs) or tri-specific killer engagers (TriKEs). It consists of a single-chain variable fragment (scFv) targeting activating receptors on NK cells (such as CD16 and NKG2D), another scFv targeting tumor specific antigens, with additional domains that may further boost NK effector functions to enhance the therapeutic effects on cancers [13] (Figure 3).

BiKEs and TriKEs were first developed for hematologic cancers and generate positive outcomes. For instance, a TriKE named 161,533 TriKE, consisting of one scFv against CD16, another scFv against tumor antigen (TA) CD33, and an IL-15 in between as a linker, effectively triggers NK recognition of neoplastic mast cells, which is normally resistant to NK-mediated immunity, as well as augments NK cell activation, degranulation and inflammatory cytokine production [159,160]. The success in hematologic cancers leads to the development of BiKEs and TriKEs for solid tumors. BiKEs and TriKEs targeting tumor antigens HER2 [161,162,163], EGFR [164], EpCAM [165,166], B7H3 [162] and CD133 [167,168] exhibit powerful therapeutic effects against corresponding solid tumors by boosting NK immune responses in preclinical studies. As such, AFM24, an EGFR x CD16A BiKE, is currently entering phase I/II studies in advanced solid cancers (Table 1) [164].

Moreover, Bogen et al. [169] proposed an innovative approach to simultaneously block EGFR and PD-1/PD-L1 signalings, and meanwhile to potentiate NK-mediated ADCC against EGFR^+^PD-L1^+^ cancer cells, with a tri-specific anti-EGFR×CD16a×PD-L1 antibody. In this study, they chose an Fc-based format to elongate the half-life of this tri-specific antibody, thus overcoming the limitation of short half-life of BiKEs and TriKEs.

Furthermore, Gauthier et al. recently developed a novel TriKE (NKp46/Fc/EGFR) targeting both NKp46 and CD16 to activate NK cells against lung carcinoma. This NKp46/Fc/EGFR TriKE shows remarkable advantages in augmenting NK infiltration in tumor tissue and promoting their anti-cancer efficacy over anti-EGFR mAb therapies such as cetuximab. Here, NKp46 is selected as a target since other activating receptors including NKp30, NKp44 and NKG2D are usually downregulated in breast cancer, lung carcinoma and AML. Notably, NKCE targeting NKp46 shows nearly 100 times higher affinity to NK cells and initiates far more potent and specific stimulation for NK activation as compared with targeting CD16 [170].

### 4.3. Strengthening NK Effector Functions

#### 4.3.1. Cytokine Therapies

Cytokine therapy is the first immunotherapy used for cancer patients, with IFN-α and IL-2 approved by FDA as treatments for several malignant cancers, including lymphoma, hairy cell leukemia, melanoma and renal cell carcinoma [171,172,173,174,175]. Both cytokines are important activators for NK-mediated immune responses.

Type I IFN (IFN-α/β) stimulates NK cell activation, improves IFN-γ production and enhances NK cytotoxicity against target cells [176,177]. More importantly, Type I IFN increases the production of IL-15 by accessory dendritic cells, which is crucial for NK maturation, activation, proliferation and cytokine production [178].

Although IL-2 is not indispensable for NK maturation and activation, it plays an essential role in IFN-γ production by NK cells, and thus enhances the immunoregulatory effects and cytotoxicity against “missing-self” targets of NK cells [179]. Despite its positive roles in strengthening NK cell immune responses, IL-2 is notorious for inducing Treg cell expansion. A study of high-dose IL-2 therapy on melanoma patients showed Treg is the most proliferative cell type after IL-2 administration, and the accumulation of Treg is correlated with poor prognosis [180]. A recently developed IL-2 agonist, NKTR-214, overcomes the above dilemma to stimulate CD8^+^ T cell and NK cell proliferation and activation without increasing Treg cells [181]. However, high-dose IL-2 administration may induce severe adverse effects, such as vascular leakage syndrome, cardiovascular disease and even neurologic toxicity [182,183].

Similar to IL-2, IL-15 also plays a pivotal role in NK maturation and acquisition of cytotoxic function. Clinical results showed that recombinant IL-15 monotherapy is well-tolerated and effectively activates NK cells, γδ cells and CD8 memory cells in patients with metastatic melanoma and renal cell carcinoma. More importantly, IL-15 administration has no significant impact on the number of Treg cells [184]. It also serves as an important adjuvant therapy for adoptive NK therapy to enhance NK effector function and to stimulate their proliferation and survival in vivo [185]. Additionally, IL-15 plays a principal role in ex vivo priming to enhance NK cell expansion and cytotoxicity prior to NK cell adoptive therapy, which will be discussed in the following section on adoptive NK cell therapies.

#### 4.3.2. Blocking Immunosuppressive Signalings

As described earlier, TGF-β severely impairs NK cell recruitment, activation and cytotoxic functions in TME. Consequently, targeting TGF-β signaling represents a viable approach to restore NK-mediated anti-cancer effects in solid tumor.

Our previous study demonstrated that blocking TGF-β/Smad3 signaling substantially rehabilitates NK cell-mediated immune response against LLC lung carcinoma and B16F10 melanoma [186]. Additionally, latency-associated peptide (LAP)/TGF-β complex on Tregs and other suppressive immune cells also serve as a promising therapeutic target. Anti-LAP mAb effectively enhances T and NK cell accumulation and their granzyme B production in mouse melanoma model [187]. Meanwhile, Ravi et al. [188] designed bifunctional antibody-ligand traps *a*-CTLA4-TGFβRII and *a*-PDL1-TGFβRII to simultaneously impair immune checkpoint CTLA4/PDL1 and TGF-β inhibitory signalings, which effectively reshapes the immunosuppressive microenvironment. As a result, it greatly reduces the number of Treg cell and strengthens the cytotoxicity of CD8^+^ T. This strategy is currently being tested in advanced solid tumors (including breast cancer, prostate cancer, head and neck squamous cell carcinoma) in multifarious clinical trials (NCT03620201, NCT04958434, NCT04633252 and NCT04428047). The role of NK cell-mediated immunity in this therapy will also be evaluated accordingly.

Another notorious immunosuppressive metabolite that protects tumors from immune response is adenosine. Blocking adenosine signaling with A2A adenosine receptor antagonists or by genetic modification largely reinforces NK cell terminal maturation, tumor infiltration, effector functions as well as IFN-γ and TNF-α productions [189,190] (Figure 3). Preclinical results also showed that the combination therapy of A2A receptor inhibitor and anti-PD-1 mAb effectively suppresses tumor metastasis as compared with either monotherapy [191]. Similar strategy combining A2A receptor inhibitor with ICIs (mainly anti-PD-1 mAb and anti-PD-L1 mAb) to prevent solid tumor progression and metastasis is currently under clinical investigations in multiple solid tumors (NCT02655822, NCT04895748 and NCT03207867).

#### 4.3.3. Immunomodulatory Therapies

Immunomodulatory drugs (IMiDs), for instance, thalidomide, lenalidomide and pomalidomide, serve as the first-line treatment in hematologic malignancies such as multiple myeloma and myelodysplastic syndromes by both directly inhibiting tumor cell growth and improving immune cell anti-cancer activities. Recent study showed that IMiDs enhance T cell and NK cell cytotoxicity and granzyme B production either through a zeta-chain-associated protein kinase-70 (Zap-70)-dependent or a CRBN/IKZF3-dependent pathway [192] (Figure 3). IMiDs also indirectly enhance NK cell activation and functions through increasing the productions of IL-2 and IFN-γ by T helper cells and dendritic cells in a SOCS1-dependent manner [193]. Importantly, IMiDs further promote cancer-killing activities of immune cells while antagonizing MDSC-mediated immune suppression when combined with ICIs [194].

Glycogen synthase kinase 3 (GSK-3) inhibitors are another rising new therapy to improve target recognition and cytotoxic functions of NK cells. Mechanistically, GSK-3 inhibition increases the expression of LFA on NK cell, which facilitates NK physical interaction with target cells as well as its subsequent activation. Additionally, suppression of GSK-3 pathway upregulates TNF-α production by NK cells through activation of NF-κB signaling [195].

Furthermore, proteasome inhibitor bortezomib can be used to strengthen NK cytotoxicity in a different manner. Bortezomib increases NK cancer killing activity by elevating FasL- and TRAIL-mediated tumor apoptosis as well as perforin/granzyme-mediated cytotoxicity [196,197] (Figure 2).

### 4.4. Adoptive NK Cell Therapies

#### 4.4.1. Cell Sources

Different from cytotoxic T cells-based therapies, autologous NK adoptive transfer therapy fails to control melanoma and renal cell carcinoma progression despite the fact that the transferred NK cells persist in circulation for weeks or even months [198]. Conversely, NK cells exert significant alloreactive cytotoxicity against cancer cells after haploidentical stem cell transplantation in leukemia patients. Subsequent clinical studies further confirmed that immunotherapy with haploidentical NK cell infusion is well-tolerated and can result in complete remission in high-risk AML patients [199,200]. The potent graft-versus-leukemia (GVL) effects with reduced graft-versus-host disease (GVHD) of allogeneic NK infusion therapy should be accredited to the lack of KIR-mediated inhibitory signaling as well as the elimination of host antigen-presenting cells. These encouraging findings pave the way for a bright future for subsequent NK-cell based immunotherapies [201,202]. Moreover, significant in vivo NK cell expansion is observed in patients pretreated with high-dose immunosuppressant cyclophosphamide and fludarabine (Cy/Flu), which may be associated with the remarkable increase in endogenous IL-15 level after intensive lymphocyte depletion [203].

While in situations of limited availability of peripheral blood NK cells, “off-the-shelf” products such as NK cell lines, NK cells derived from umbilical cord blood (UCB) and induced pluripotent stem cells (iPSCs), would serve as potential and feasible alternatives.

The most commonly used NK cell line is NK92, which shows high cytotoxicity to a wide spectrum of cancer cells. However, due to their genetically instability, NK92 cells must be irradiated before infusion, which in turn impairs their capability of proliferation and limits their persistence in vivo [204].

NK cells account for 10% of lymphocytes in peripheral blood, while they account for 30% in UCB, thus making UCB an abundant source of NKs for adoptive therapy [205]. Of note, KIR haplotype largely influences the therapeutic effects of UCB-NK therapy [206,207]. Although only limited preclinical studies showed positive outcome of UCB-NK therapy, it is now being tested in several clinical trials in various hematologic malignancies (NCT02727803, NCT01729091 and NCT02280525), as well as solid tumors (Table 2).

The recently developed iPSC-derived NK cells (iPSC-NKs) may serve as a superior candidate for NK cell therapy, which is more accessible and genetically stable for large-scale manufacture, quality control, and genetic engineering. Cichocki et al. [208] reported that iPSC-NKs possess similar characteristics to peripheral blood NK cells, with impressive potential to expand 1 × 10^6^-fold ex vivo, while secreting high levels of inflammatory cytokines and exerting high cytotoxicity against diverse cancer cells. When combined with anti-PD-1 mAb, iPSC-NK cells further promote immune responses via facilitating T cells recruitment and activation, which helps to overcome the barrier of checkpoint blockade resistance. This up-and-coming approach is now entering phase 1 clinical trial (Table 2) [208].

#### 4.4.2. Genetic Engineering of NK Cells

The application of chimeric antigen receptors (CARs) in cytotoxic T cells considerably enhances T cell recognition and adhesion to target tumor cells and their subsequent activation, which accounts for the tremendous success in treating various hematologic malignancies. However, the major concern for CAR-T therapy is the severe side-effects including cytokine release syndrome, graft-versus-host disease and neurotoxicity [209]. On the other hand, despite favorable results of NK cell therapy in hematologic malignancies, its recruitment, recognition and activation are severely blunted in solid tumors, as described previously. Therefore, CAR-NK and other genetic-engineered NK cell therapies may represent alternative strategies to provide potent immunosurveillance while circumventing the limitations of CAR-T therapies.

CAR-NKs are normally derived from NK92, UCB-NKs and iPSC NKs. They are genetically modified to express synthetic chimeric receptors constructing with an extracellular antigen-binding domain (scFv of tumor-specific antigens), a transmembrane domain and an intracellular signaling domain containing one or several stimulatory molecules, such as CD3ζ chain, CD28 or 4-1BB [210] (Figure 3). As mentioned previously, CD27^high^ NK cell represents as a highly cytotoxic phenotype. Turaj et al. [211] recently discovered that stimulating this subset of NK cells with anti-CD27 mAb promotes the accumulation of myeloid cell activation and infiltration in the TME through releasing IFN-γ and chemokines. Therefore, CD27 may also serve as a potential costimulatory receptor that can be incorporated into the CAR gene to promote NK effector functions.

In spite of the limited availability of specific tumor antigen in solid tumors, such as HER2 and EGFR (or EGFR variant III) on breast cancer and glioblastoma [212,213,214,215,216], CS1 and CD138 on myeloma [217,218], GD2 on neuroblastoma [219,220] or NKG2D ligand on ovarian cancer and osteosarcoma [221,222], the application of CAR-NK therapies achieves impressive outcomes in the preclinical studies, without causing severe adverse effects like those by CAR-T therapies. The safety and effectiveness of CAR-NK therapies are now tested in several phase 1 and phase 2 trials in solid tumors (Table 2).

Apart from optimizing NK recognition and activation by solid tumors, genetic engineering endows persistent NK therapy via incorporating IL-15 into CAR gene, similar to the design of utilizing human IL-15 as a crosslinker in BiKEs and TriKEs [223]. Other functional modifications include preventing TGF-β- and glucocorticoids-induced suppressions on NK cell cytotoxic functions, as glucocorticoids reduce the production of IFN-γ by splenic NK cells through upregulating the expression of PD-1 [224]. This is achieved by genetically engineering to disrupt TGF-β receptor 2 and glucocorticoid receptor [224], express dominant negative TGF-β receptors [225], or deplete Smad3, the downstream transcriptional factor of TGF-β signaling [226] (Figure 3).

#### 4.4.3. Ex Vivo or In Vivo NK Cell Expansion and Activation

Because of the large amount of NK cells required for a single dose infusion, ex vivo NK cell expansion is a prerequisite for adoptive NK cell therapies. Multiple cytokines have been used for NK ex vivo expansion, including IL-2, IL-12 IL-15, IL-18 and IL-21, with or without feeder cells genetically engineered to express cytokines and co-stimulatory molecules. For instance, Wagner et al. [227] proposed a two-phase expansion protocol consisting of IL-15-stimulated early-stage expansion and subsequent IL-21-boosted activation. Their result showed the addition of short-term IL-21 stimulation effectively boosts NK cell cytotoxicity as compared with IL-15 stimulation alone. Fujisaki et al. [228] proved that the use of feeder cell K562 expressing IL-15 and 4-1BB alone, can induce a 21.6-fold expansion of highly cytotoxic peripheral blood-derived NK cells without influencing CD3^+^ lymphocytes.

Another approach to promote NK cell expansion, persistence and function in vivo is by genetically transducing IL-2 or IL-15 gene into NK cells, which also helps to circumvent the adverse effects induced by cytokine administration [229,230,231].

## 5. Conclusions and Perspectives

NK cells serve as a superior tool for immunotherapy with high anti-cancer efficacy and relatively low adverse effects. It benefits from its intrinsic recognition capability thereby independent of antigen presentation, and provides definitive protection against nascent metastasis, cancer recurrence and relapse [148]. The great preclinical success and tremendous advances in NK-based immunotherapies provide a promising future for patients with hematologic cancers.

However, the solid tumor microenvironment remains a big challenge for NK-based immunotherapies. One of the main challenges for immunotherapy in solid tumors is the very limited available tumor specific antigens due to the heterogeneity of solid tumors with diverse gene profiles and mutations signature. Combination of multiple immunotherapies for targeting divergent tumor antigens may help to tackle this problem and thus providing superior NK-mediated immunosurveillance without the risk of inducing NK exhaustion by overwhelmingly intense monotherapy [232].

Another obstacle is the aberrant vasculature, which largely limits the accumulation of cytokine, antibodies and NK cells in solid tumors. The burgeoning nanodrug delivery system enables tissue-specific delivery, reducing the accumulation of cytokine or antibodies in circulation, thus consequently widening their dosage windows to provide safer and more effective immunotherapies [233].

Notwithstanding, each therapeutic method has its own merits and drawbacks. The application of cytokine monotherapy is significantly limited by the rather narrow therapeutic window of systematic administration and short half-life [66].

Compared with CAR-NKs, BiKEs and TriKEs are more easily manufactured and cost-effective. However, BiKEs and TriKEs suffer from short half-life and structural instability due to the lack of Fc region [169]. Similarly, ICIs also have relatively short half-life. More importantly, the low bioavailability of antibody-based therapies such as KEs and ICIs owing to the low tumor to blood ratio severely restricts their clinical efficacy [234]. Fortunately, the aforementioned recent nanotechnology enables tumor-specific delivery to circumvent such obstacle [233]. Although KEs and ICIs can be more easily dosed and controlled thanks to their short half-lives and incapability to expand in vivo, these properties also largely limit their therapeutic effects and increase cost. Therefore, incorporating killer engager and ICI genes into CAR-NKs to produce KEs or ICIs in vivo may represent a novel strategy to overcome this hurdle and to further augment CAR-NK activation and cytotoxic function against cancer cells.

Although NK cells consist of several subpopulations with divergent functions, ex vivo expansion prior to adoptive transfer specifically manufactured highly cytotoxic NK populations, and genetic engineering additionally augments their activation and cytotoxic functions [235]. Compared with CAR-T therapies, CAR-NK therapies demonstrate multiple remarkable advantages, such as enhanced antibody-dependent cell cytotoxicity (ADCC) when combined with antibody therapies, “off-the-shelf” feasibility owing to the benefit of allogeneic transplantation, lower risk of graft-versus-host disease and reduced side-effects [9]. In terms of tumor antigen loss or antigen-low escape occurred in CAR-T therapies, CAR-NK therapies still benefit from their intrinsic recognition capacity via NCRs and NKG2D [236]. While NK cells suffered from greater difficulties in large-scale expansion and genetic engineering as compared to T cells [232], transfection methods optimized recently taking advantages of AVV vector, transposons and CRISPR/Cas9 technique largely facilitate the manufacture of CAR-NK cells [237].

The immense advances in multi-omics technologies shed lights on novel regulatory mechanisms of NK-mediated immunity and their crosstalk with the TME [238]. Together with the emerging novel drug delivery techniques, exciting developments of immunotherapies specific for solid tumors can now fully exploit both the anti-cancer effects and the immunoregulatory prowess of NK cells to reshape the immunosuppressive microenvironment [239]. With an exceeding number of therapies now entering clinical trials every year, a new era full of bright prospect NK cell-targeted immunotherapies is to be anticipated.

## Figures and Tables

**Table 1:** Selected ongoing clinical trials targeting NK recognition and activation.

Target	Therapy	Clinical Trials Identifier	Eligibility
ADAM10, ADAM17	ADAM10 and ADAM17 inhibitor (INCB7839)	NCT04295759 (Phase 1)	Gliomas
CD16	EGFR x CD16A BiKE (AFM24) with autologous NK therapy	NCT05099549 and NCT04259450 (Phase 1 and 2)	EGFR^+^ cancers
KIR	Lirilumab, combined with anti-PD1 (ivolumab) and anti-CTLA-4 mAb (ipilimumab)	NCT03203876 (Phase 1)	Advanced and/or metastatic solid tumors
	Lirilumab, combined with anti-PD1 mAb (nivolumab)	NCT03341936 (Phase 2)	Squamous cell carcinoma of the head and neck
NKG2A	Monalizumab, combined with anti-HER2 mAb (trastuzumab)	NCT04307329 (Phase 2)	HER2^+^ breast cancer
	Monalizumab, combined with anti-PD-L1 mAb (durvalumab)	NCT04145193 and NCT05061550 (Phase 2)	Non-small cell lung cancer
PD-1	anti-PD-1 mAb, combined with chemotherapy	NCT03983057 (Phase 3)	Pancreatic Cancer
	anti-PD-1 mAb, combined with chemoradiotherapy	NCT04301557 (Phase 2)	Advanced colorectal cancer
CTLA-4	Fc-engineered IgG1 anti-CTLA-4 mAb (AGEN1181)	NCT03860272 (Phase 1 and 2)	Advanced solid tumors
	Ipilimumab, combined with anti-PD1 (nivolumab) and anti-LAG3 mAbs (relatlimab)	NCT04080804 (Phase 2)	Squamous cell carcinoma of the head and neck
LAG-3	Relatlimab, with or without anti-PD-1 mAb (nivolumab)	NCT01968109 (Phase 1 and 2)	Solid tumors
	Relatlimab, with or without anti-PD-1 mAb (nivolumab)	NCT03610711 and NCT03662659 (Phase 1 and 2)	Advanced esophagogastric cancer
TIM-3	BGB-A425, combined with anti-PD1 mAb (tislelizumab)	NCT03744468 (Phase 2)	Advanced solid tumors
	anti-PD-1/anti-TIM-3 bispecific antibody (AZD7789)	NCT04931654 (Phase 2)	Advanced and/or metastatic solid tumors
TIGIT	Ociperlimab, combined with anti-PD1 mAb (tislelizumab)	NCT04746924 (Phase 3)	Non-small cell lung cancer
	anti-TIGIT/anti-PD-L1 bispecific antibody (HLX301)	NCT05102214 (Phase 1 and 2)	Advanced or metastatic solid tumors

**Table 2:** Selected ongoing clinical trials using adoptive NK cell therapies.

Cell Source	Gene Engineering	Combined Therapy	Clinical Trials Identifier	Eligibility
Autologous NK cells	N/A	Gemcitabine and carboplatin, with or without cetuximab	NCT04872634	Non-small cell lung cancer
Autologous NK cells	N/A	Chemotherapy 5-FU and cisplatin	NCT05040438 (Phase 2)	Advanced hepatocellular carcinoma
Donor-derived NK cells	N/A	N/A	NCT04162158 (Phase 1 and 2)	Advanced hepatocellular carcinoma
Donor-derived NK cells	N/A	Anti-GD2 mAb (hu3F8)	NCT02650648 (Phase 1)	Neuroblastoma
Donor-derived NK cells	N/A	N/A	NCT04616209 (Phase 1 and 2)	Non-small cell lung cancer
Donor-derived NK cells (FATE-NK100)	N/A	Combine with anti-EGFR mAb (cetuximab) for advanced EGFR1+ solid tumors; combine with anti-HER2 mAb (trastuzumab) for advanced HER2+ solid tumors	NCT03319459 (Phase 1)	Advanced solid tumors
Donor-derived NK cells	N/A	Allogeneic HCT 7 days prior to NK cell infusion	NCT02100891 (Phase 2)	Solid tumors
iPSC-derived NK cells (FT500)	N/A	Monotherapy or in combination with anti-PD1 mAb (nivolumab/pembrolizumab) or anti-PD-L1 mAb (atezolizumab)	NCT03841110 (Phase 1)	Advanced solid tumors
UCB-derived NK cells	N/A	Chemotherapy cyclophosphamide and etoposide	NCT03420963 (Phase 1)	Relapsed or refractory solid tumors
N/A	Irradiated high-affinity CAR targeting PD-L1 (PD-L1 t-haNKs)	Anti-PD-1 mAb (pembrolizumab) and IL-15 superagonist (N803)	NCT04847466 (Phase 2)	Gastric or head and neck cancer
NK92 cells	Express CAR targeting Robo1	N/A	NCT03941457 and NCT03940820 (Phase 1 and 2)	Pancreatic cancer and other solid tumors
NK92 cells	Express CAR targeting HER2	N/A	NCT03383978 (Phase 1)	Recurrent HER2-positive Glioblastoma
UCB-derived NK cells	Delete TGF-BetaR2 and NR3C1	N/A	NCT04991870 (Phase 1)	Recurrent glioblastoma
“off-the-shelf” NK cells	Express CAR targeting HER2	N/A	NCT04319757 (Phase 1)	Advanced or metastatic HER2-expressing solid tumors
iPSC-derived NK cells	Express high-affinity non-cleavable CD16 (FT516)	Fc-optimized humanized IgG1 anti-B5-H3 mAb (enoblituzumab)	NCT04630769 (Phase 1)	Ovarian cancer
		Anti-PD-L1 mAb (avelumab)	NCT04551885 (Phase 1)	Advanced solid tumors
iPSC-derived NK cells	Delete CD38, express an IL-15 receptor alpha fusion protein and a high-affinity non-cleavable CD16	Multiple monoclonal antibodies	NCT05069935 (Phase 1)	Advanced solid tumors
